# A functional near-infrared spectroscopy study on the prefrontal correlates of cognitive offloading via a personal knowledge assistant

**DOI:** 10.1038/s41598-023-39540-5

**Published:** 2023-08-25

**Authors:** Christoph Geissler, Paula Gauselmann, Christian Jilek, Heiko Maus, Christian Frings, Tobias Tempel

**Affiliations:** 1 Department of Cognitive Psychology, University of Trier, 54286 Trier, Germany; 2 Institute for Cognitive and Affective Neuroscience (ICAN), University of Trier, Trier, Germany; 3 Ludwigsburg University of Education, Ludwigsburg, Germany; 4 German Research Center for Artificial Intelligence (DFKI), Kaiserslautern, Germany; 5 RPTU Kaiserslautern-Landau, Kaiserslautern, Germany

**Keywords:** Psychology, Human behaviour

## Abstract

The saving of previously encoded information boosts both memory for subsequent information (saving-enhanced memory; SEM) as well as cognitive performance in general (saving-enhanced performance; SEP). These findings have been replicated in a setting that involves the assistance by an intelligent software that automatically structures and saves work content in an interactive sidebar. It is assumed that beneficial effects on cognitive performance due to (automatic) saving are caused by a reduction in current workload by means of cognitive offloading. We tested this assumption by measuring neural activity in the dorsolateral prefrontal cortex (DLPFC) via functional near infrared spectroscopy (fNIRS)—once after saving and once after deleting of previously collected information that had to be recalled later-on. On a behavioral level, there was a brief benefit of saving. However, cognitive offloading became most apparent on a neural level: after saving, participants showed significantly lower activation in the right DLPFC. Also, the more participants benefited from cognitive offloading, the more they were able to re-access previously collected, saved information. Thus, fNIRS results indicated reduced mental load after saving, confirming the assumption that saving triggers cognitive offloading.

## Introduction

We live in times of what can be called a problematic information overload. Digital input from many sides (e.g., messages, e-mails, news, personalized ads, social media etc.) is constantly draining our attentional resources—mostly even without us noticing it. Too much (digital) information not only can leave us stressed and even anxious or depressed^1^ but has also been shown to have a negative impact on our cognitive abilities^2–4^. Yet, being aware of this gives us the chance to counteract information overload. In private life, we may simply switch off our phones or any given digital device. However, during working hours this is not as simple or even impossible wherever the profession involves computer interaction.

Cognitive offloading has been shown to be a promising and effective way of shielding ourselves against unwanted masses of input^5^. Even though this term has only been in the focus of research for a few years, it comprises strategies and actions that have been of great relevance to our existence for much longer. It is understood as the use of physical action that temporarily lightens cognitive demands. An action like this can either be something that externalizes internal information (e.g., writing thoughts down) or a movement that alters internal perception in a way that makes targeted information easier to process (e.g., tilting the head when looking at a rotated figure).

A modern version of cognitive offloading is (digital) saving. A study by Storm and Stone^6^ demonstrated for the first time an effect of saving-enhanced memory (SEM). In their study, participants memorized two word lists in the form of pdf files (L1, L2) before being tested first on L2 and then on L1. After studying of L1, they were either instructed to save or to not save it before closing. In the case of saving, they were able to re-access the file for later restudy before being tested on it, allowing them to temporarily offload this information onto the computer. Results showed that saving of L1 significantly enhanced memory for L2. However, this depended both on trust in the reliability of the saving process and the first list’s potential to interfere with the second one, i.e. whether the information to be held in memory in case of no saving was substantial enough to hinder subsequent encoding of new information.

The effect of SEM has been replicated several times under varying circumstances, e.g. after instructions to delete instead of not to save^7^, for finger movements^7^ and in eye gaze interaction^8^. Importantly, recent studies have further demonstrated that saving seems to not only enhance memory but cognitive performance in general. For example, Runge and colleagues^9^ adapted the original SEM paradigm in a way that in addition to memorizing and being tested on two lists, participants had to work on blocks of modular arithmetic problems—once after saving vs. deleting of L1 and again before the test of L2. Results showed an effect of saving-enhanced performance (SEP): participants solved significantly more modular arithmetic problems after saving of L1.

Based on these findings, there is no doubt that saving enables us to make better use of our limited cognitive resources. However, efficient information management involves more than just the simple act of saving, but also filtering relevant from irrelevant content, switching back and forth between tasks and meanwhile keeping track of differing information scattered all over a device. Researchers have recently developed a tool that aims at supporting the user in these challenging tasks. Based on artificial intelligence, the so-called *Semantic Desktop*^10^ or a more recent and advanced version *Context Spaces*^11^ gather, display and constantly update a representation of a user’s mental model^12^. In short, the system continuously learns from the user’s interaction with an indefinite number of so-called “things” (e.g., files, tasks, web pages etc.), each of which is assigned relevance values for a user’s different (working) contexts. For example, a certain file may be very important in one context (e.g. project A) while being totally irrelevant in another (project B). Further details about this relevance assessment can, for example, be found in Jilek et al.^13^. Based on this, the intelligent assistance software can display relevant content (e.g. previously used in or explicitly associated with a certain context) at any given time in appropriate situations or contexts, whereas “appropriate” here typically refers to the very same contexts being revisited at a later point in time or new contexts that are similar to existing ones.

In a study by Gauselmann et al.^21^, a prototype of this software was used to investigate whether relying on it in work-like scenarios would entail similar beneficial effects on cognitive performance as when saving is done by one’s own hand. Results confirmed this assumption: one experiment demonstrated how classifying which parts of digital input are currently most relevant enhances memory for this specific input. A second experiment displayed how, similar to the effect of SEM, automatic structuring and saving of content in one (work-) context by an interactive sidebar benefited performance in modular arithmetic problem solving (i.e., a new, unrelated context).

While the effect of SEM for related content has already been further investigated for underlying mechanisms^7^, it is still unclear what exact mechanisms cause beneficial effects on performance in unrelated, cognitively demanding tasks like the ones reported above. However, it is assumed that these can be attributed to a temporarily reduced working memory load due to interrupted rehearsal of information that, without saving, would have to be kept in memory.

### The present study

To affirm that the effects of sidebar-saving vs. deletion found in the study by Gauselmann et al.^21^ indeed are caused by cognitive offloading, we chose a neuropsychological approach. We used functional near infrared spectroscopy (fNIRS) to track workload related changes in prefrontal neural activity in the arithmetic task following sidebar saving vs. deletion. fNIRS optically detects changes in oxygenated hemoglobin [oxyHB] and deoxygenated hemoglobin [deoxyHB] concentrations related to changes in neural activation in outer cortical regions. Both a local rise in [oxyHB] and a local decline in [deoxyHB] can be regarded as the result of a rise in local neuronal activity. In the past, several fNIRS-studies have reported that the dorsolateral prefrontal cortex (DLPFC) is especially sensitive to changes in acute workload, both in laboratory^14–16^ and applied settings^17–20^. Here, we replicated experiment 2 of Gauselmann et al.^21^ and measured DLPFC activity during the modular arithmetic-blocks. We expected that neural activity in this area would be substantially lower when the sidebar was saved, and participants did not have to simultaneously keep in mind the facts from the inquiry while solving modular arithmetic problems.

## Results

**Figure 1:**
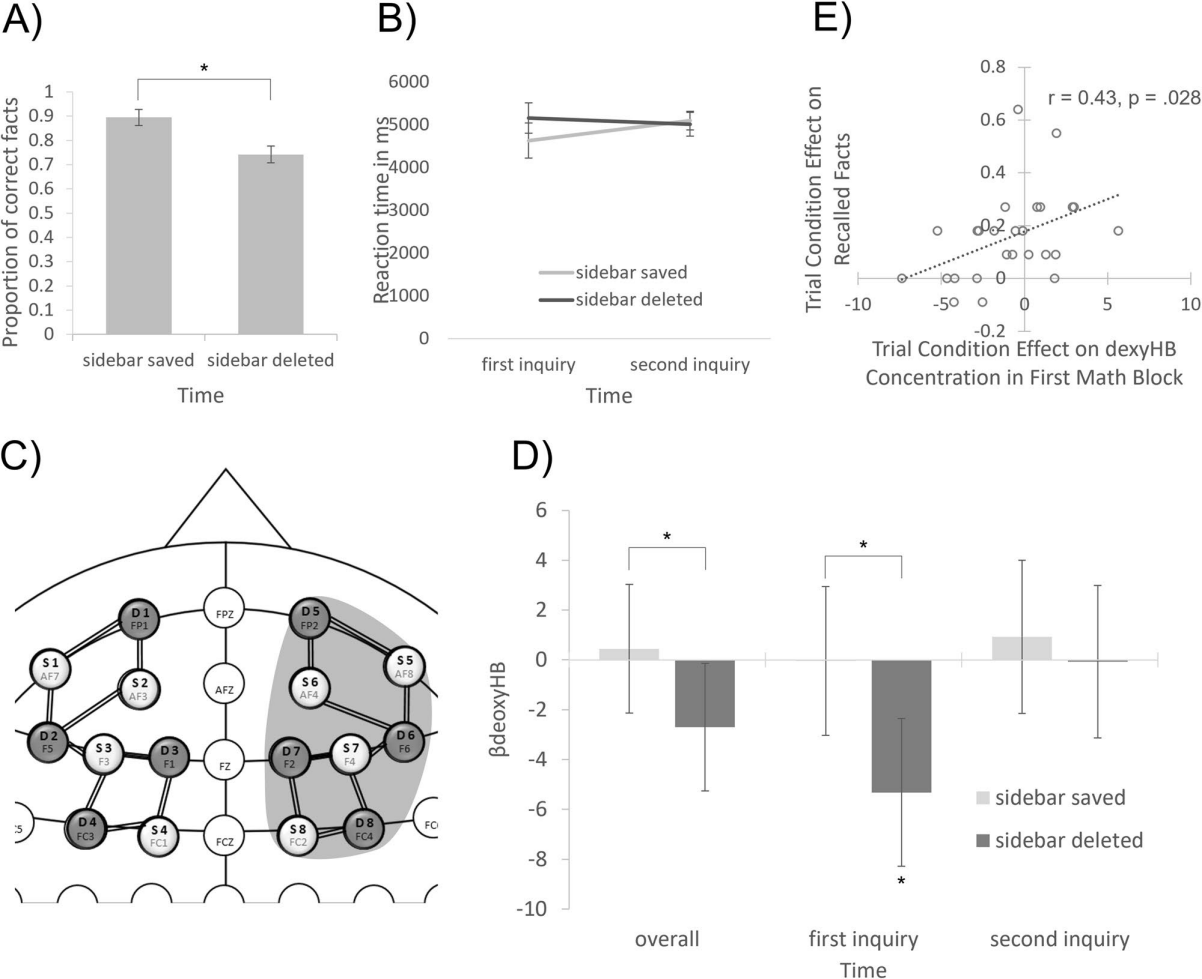
Results. Error bars in (**A**) and (**B**) represent two-times within standard errors after Morey^31^. Error bars in D represent two-times standard errors. (**A**) Correctly reported facts in the factsheet. (**B**) Mean reaction time for first five modular arithmetic problems. (**C**) Localization of hemodynamic trial condition effects in deoxyHB. (**D**) Hemodynamic results for trial condition effect in deoxyHB. (**E**) Correlation between trial condition effects on correctly recalled facts in the fact sheet and right DLPFC activation in deoxyHB.

A repeated measurement t-test with saving as independent variable and percent of correctly reported facts in the fact sheet as dependent variable revealed that significantly more correct facts were reported in the sidebar saved (89%, SD = 11) than in the sidebar deleted (73%, SD = 18) condition (*t*(25) = 4.73, *p* < 0.001, *d*_*z*_ = 1.04 see Fig. 1A). Repeated measurement ANOVAs with trial condition and time as independent factors and mean reaction time (RT) and accuracy (ACC) in arithmetic tasks respectively as dependent variables revealed no significant main effects or interactions (all *F* < 1.60, all *p* > 0.20). Because some earlier research showed an effect of cognitive offloading with only five arithmetic tasks^8^, we also looked at offloading effects on RTs and ACC in the first five modular arithmetic tasks in a separate exploratory analysis. For RTs, this analysis revealed a significant interaction between the factors trial condition and time (*F*_*1, 22*_ = 4.96, *p* = 0.037, *Ω*^2^ = 0.04, see Fig. 1B). Simple effects asnalysis revealed that RTs in the first five arithmetic tasks were significantly lower after sidebar saving (4508 ms, *SD* = 927 ms) than after sidebar deletion (5162 ms, *SD* = 1059 ms) after the first inquiry (*t*(23) = − 2.47, *p* = 0.022, *dz* = − 0.66). No other main effects or interactions reached significance in this exploratory analysis (all *F* < 3.18, all *t* < 1.01, all *p* > 0.05). None of the effects where significantly moderated by the control factor trial sequence (all *F* < 2.80, all *p* > 0.10). See Table 1 for arithmetic performance results.

**Table 1:** Arithmetic Performance.

		All trials		First five trials	
		Reaction times	Error rates	Reaction times	Error rates
First inquiry	Sidebar saved	5144.03 (690.68)	0.55 (0.18)	4634.18 (994.72)	0.55 (0.25)
	Sidebar deleted	5145.48 (807.84)	0.54 (0.23)	5162.49 (1059.4)	0.59 (0.27)
Second inquiry	Sidebar saved	5111.47 (740.07)	0.57 (0.21)	5099.09 (797.89)	0.58 (0.31)
	Sidebar deleted	5067.89 (743.6)	0.58 (0.21)	5017.23 (847.91)	0.62 (0.24)

On a neural level, one of the ROIs showed significant differences regarding sidebar saved and sidebar deleted conditions: in deoxyHB, the right DLPFC showed higher activation after sidebar deletion over both modular arithmetic blocks combined (*β*_ΔdeoxyHB_ = − 3.14, *t*_[deoxyHB]_ = − 3.57, *p* = 0.003), as well as in the first block alone (*β*_ΔdeoxyHB_ = − 5.28, *t*_[deoxyHB]_ = − 3.96, *p* = 0.002). Closer inspection showed that these effects were driven by a significant increase of right DLPFC activity in the first block of the sidebar deleted condition (*β*_deoxyHB_ = − 5.32, *t*_[deoxyHB]_ = − 3.59, *p* = 0.003). See Fig. 1C and D for significant functional hemodynamic results. See Table 2 for all functional hemodynamic results.

**Table 2:** Full functional hemodynamic results.

Contrast	ROI	Blood type	Beta (SE)	t	*p*
[ΔHB Deleted Overall] – [ΔHB Saved Overall]	Left DLPFC	oxyHB	− 0.44 (1.76)	− 0.25	0.946
		deoxyHB	− 1.51 (0.78)	− 1.95	0.135
	Right DLPFC	oxyHB	0.93 (1.78)	0.52	0.804
		deoxyHB	− 3.14 (0.88)	− 3.57	0.003
[ΔHB Deleted First Inquiry] – [ΔHB Saved First Inquiry]	Left DLPFC	oxyHB	5.03 (2.78)	1.81	0.146
		deoxyHB	− 2.88 (1.20)	− 2.4	0.073
	Right DLPFC	oxyHB	1.65 (2.83)	0.58	0.804
		deoxyHB	− 5.28 (1.33)	− 3.96	0.002
[ΔHB Deleted Second Inquiry] – [ΔHB Saved Second Inquiry]	Left DLPFC	oxyHB	− 5.91 (3.06)	− 1.93	0.135
		deoxyHB	− 0.15 (1.31)	− 0.11	0.946
	Right DLPFC	oxyHB	0.21 (3.09)	0.07	0.946
		deoxyHB	− 1.00 (1.47)	− 0.68	0.804

All *p*s represent Benjamini–Hochberg^29^ corrected significances.

In addition, there was a significant correlation between the trial condition effect on the number of correctly filled in facts in the memory test and the trial condition effect on deoxyHB concentration in the first arithmetic block (*r* = 0.43, *p* = 0.028, see Fig. 1E). The greater the reduction in neural activity due to sidebar saving after the first inquiry, the greater the benefit of sidebar saving on fact recall. Thus, the brain activity of participants that were able to fill in more facts indicated stronger cognitive offloading as compared to participants that filled in fewer facts. There were no significant correlations of memory performance with right DLPFC activity overall or in the second inquiry and no significant correlations between trial condition effects on right DLPFC activity and performance in the first five trials of arithmetic task after the first inquiry, *r* < 0.36, *p* > 0.08.

## Discussion

The aim of the present study was to replicate and expand previous insights on how automatic saving by intelligent assistance software in form of an interactive sidebar enhances cognitive performance when switching back and forth between tasks. Being specifically interested in whether beneficial effects are caused by cognitive offloading, we used fNIRS in order to compare DLPFC activity after saving to activity after deleting contents of a previous work context. Behavioral results showed that participants filled in significantly more correct facts after sidebar saving.

That more correct facts had been filled in after sidebar saving corresponds to results from Gauselmann et al.^21^. Since, in saved trials, participants were allowed to use their previous notes and work progress stored in the sidebar for filling in the fact sheet, it was to be expected that, accordingly, more correct facts were recalled as compared to when sidebar contents had been deleted. This finding once more demonstrates one possible practical scenario in which auto save might prove very useful, since the not-so-simple act of gathering and storing information by one’s own hand would not only require time and effort but also part of one’s valuable cognitive resources.

While no beneficial effect of saving was found on overall performance in solving modular arithmetic problems, an exploratory analysis of only the first five arithmetic tasks following each inquiry revealed that participants reacted significantly faster after sidebar saving than after sidebar deletion following the first inquiry. This pattern of effects fits both earlier findings of Gauselmann et al.^21^, who found effects of sidebar saving only after the first of two inquiries, and Runge et al.^8^, who found effects of cognitive offloading on arithmetic performance within five trials after the offloading manipulation. Potentially, the first trials of a new task disproportionally profit from cognitive offloading of previous task content. However, further research is necessary to confirm this. Additionally, it is possible that typical beneficial effects of saving could not be replicated due to an overall notably low performance in solving modular arithmetic problems. While participants in the studies by Runge et al.^8^ and Gauselmann et al.^21^ solved on average between 64% (after not saving) and 68% (after saving) of the mathematical problems, participants here merely solved a bit more than 50% (both after not saving and saving). Potentially, even without additional load, the modular arithmetic problems might have been to hard to solve for a significant portion of participants, which might have partially masked any potential offloading effect. Admittedly, it is not entirely clear what has led to such low performance. Several procedural details that differ between our study and previous ones might be a reason. However, it is important to note, that the low performance per so cannot explain differences between saved and deleted conditions so that the main conclusions drawn here are not endangered by the overall low performance of our participants. Nonetheless, on a neural level, results revealed overall significantly lower activation in the right DLPFC during modular arithmetic problem solving after sidebar saving. This effect was driven by significantly lower right DLPFC activation during arithmetic tasks following saving after the first inquiry. In contrast, following the second inquiry, no effect of sidebar saving vs. deletion on DLPFC activation during arithmetic tasks was found. This pattern of neural effects fits both earlier behavioral results by Gauselmann et al.^21^, who in the identical task found positive effects of sidebar saving on mathematical performance only after the first inquiry, and the effects in our own exploratory analysis of behavioral data that revealed offloading effects in the first five arithmetic tasks following specifically the first inquiry.

That sidebar saving goes along with significantly reduced right DLPFC activity during subsequent arithmetic tasks fits well with the notion that beneficial effects of cognitive offloading on current task execution are mediated by reduced working memory load^8^. The DLPFC has a crucial role in working memory processes^22,23^. In particular, it has been assumed that the DLPFC is tasked with upholding, updating and manipulating task relevant information^24^. In the sidebar not saved condition of our paradigm, this would encompass critical information from the previous inquiry as well as parameters of the current arithmetic task. On the other hand, in the sidebar saved condition only parameters of the current arithmetic task would have to be held up in the DLPFC, leading to a relative reduction in load and consequently activation. This effect might be observed mainly in the first inquiry, because in the second inquiry many facts had already been filled in and didn’t have to be actively upheld, leaving not much room for further offloading^21^. While this was mainly visible in a significant increase of [deoxyHB] in the sidebar-deleted condition after the first inquiry, it is important to state that only the relative differences in [deoxyHB] between the save and deleted conditions can be interpreted with relative certainty in our design. The reason for this is, that we did not carry out any measurements at rest and thus cannot make any statements about absolute decreases or increases in neural activity. Importantly, the relative differences in [deoxyHB] between conditions clearly point towards less activity in the saved condition and consequently comparatively lower load.

There was also a direct relation between neural and behavioral effects in our design: a correlational analysis revealed that participants who benefitted more from sidebar saving with regards to correctly recalled facts, also showed a greater decrease in right DLPFC activity during the first arithmetic block after sidebar saving compared to sidebar deletion. This effect might be mediated by more proficient usage of the semantic desktop. Thus, participants, that relied more on saving facts in the sidebar might have benefitted twice: first during the arithmetic tasks, in which they experienced lower cognitive load and again while filling out the fact sheet during which they could more easily transfer facts from the inquiry. Notably, we did not find any direct correlation between the significant neural and behavioral offloading effects in modular arithmetic (all |r|< 0.10, all *p* > 0.656). A probable cause for this might be that the generally weak behavioral offloading acted as a limiting factor for a possible correlation.

However, even though the interpretability of the reported results is somewhat limited due to the fact of the overall low cognitive performance, the significant change in frontal brain activity after saving provides an important indication of reduced memory load.

Overall, the findings of the present study demonstrate that, in times of information overload, AI-based saving is a promising tool that not only can help us organize our work but also has the potential to boost performance in secondary tasks. The reduced DLPFC activity that goes along with these performance benefits suggests that cognitive offloading helps reduce acute working memory load and, thus, allows us to focus more resources on the currently relevant task.

## Methods

### Participants

26 participants were included in the study (22 female; mean age = 25.4, SD = 6.11), in which they participated either for course credit or a monetary reward of 15 €. G*Power (v3.1.9.7^25^) sensitivity analysis for one-sample t-test indicated a required medium effect size of d = 0.57 (two-tailed) when alpha was set to 0.05 and power to 0.80. All participants stated normal or corrected-to-normal vision; no participant stated any history of neurological disease. Participants gave written informed consent to participation as well as publication of anonymized data before examination. The study was conducted in accordance with the Declaration of Helsinki (World Medical Association, 2003). Furthermore, the local ethical review committee at the University of Trier (Ethik-Kommission des Senats der Universität Trier) evaluated and approved the study (Decision to motion 72/2018, 11.12.2018).

### Design

This study had a 2 (trial condition: sidebar saved vs. sidebar deleted) × 2 (time: first inquiry vs. second inquiry) within-subjects design. Additionally, trial sequence (saving first trial vs. saving second trial) was employed as a between-subjects control factor.

### Material

The experiment was conducted on Windows 64-bit machines with a Java 8 backend and two HTML5 frontends: one as a web application in the browser and one as a permanently visible sidebar (width: 10 cm, no overlap with browser). We used a pre-configured version of Opera with three additionally installed web extensions storing activities in the backend: one documenting browsing behavior (invisible), one (visible) interactive marker pen for highlighting and storing information from browsed pages and one (visible) interactive tab closing button. Participants had a viewing distance of approximately 60 cm and made inputs via a standard German QWERTZ keyboard and a computer mouse. 60 modular arithmetic problems^26^ were randomly chosen from a pre-tested set of 80 items^8^. This type of arithmetic has been shown to indicate working-memory capacity^27^. Fact sheets were adopted from Gauselmann et al.^21^: one about a country (Laos) and one about a person (Simón Bolívar). Both sheets consisted of eleven topic-related questions (e.g., birth rate and form of government in Laos or Bolívar’s place of birth).

### Procedure

Participants were tested individually in a dimly lit room. Instructions were given both verbally and via screen. The experiment consisted of two trials (one saved trial, one deleted trial), each consisting of two rounds following the same order: inquiry, modular arithmetic problems, filling in a fact sheet. After participants were initially presented with a fact sheet containing questions about a certain topic, they were instructed to search for information that would later help them answer these questions. While doing so, participants were encouraged to make use of the sidebar that would allow them to highlight and store important information. The crucial manipulation took place when, at a seemingly random point, they were interrupted by another task (modular arithmetic problems) prior to which they were informed whether their current work progress would be saved or not. In deleted trials, participants received a note stating that all previously collected content in the sidebar would be lost. In saved trials, the note stated that all content would be saved and available to later help fill in the fact sheet. Subsequent performance in solving modular arithmetic problems was the variable of interest, with better performance indicating potential benefits of prior cognitive offloading via saving of the sidebar.

All participants started with a practice trial, accompanied by the experimenter. First, concept and features of the sidebar were demonstrated. It consisted of three main parts: (1) A button “context selection” served for switching between tasks, which were referred to as contexts. Upon clicking on the button, one could select the specific context. (2) Second, the sidebar contained all “contents of a current context”, which was displayed as a list of web pages and notes that were either added automatically, by roaming through different content, or manually, either by adding it from the displayed list of activities in a context or by making use of a marker pen. Additionally, notes could be taken in a small text box by clicking on a notepad icon next to each listed web page. (3) The third feature was an automatically updated, reverse chronological list of all activities in a context. Apart from sidebar features, participants were instructed on how to search for information. All participants started on the same web page that contained a search field that looked as if someone had already entered some keywords. These keywords could be clicked on, leading to different web pages that resembled actually existing web pages (e.g., Wikipedia). What participants didn’t know was that the search took place on a limited amount of web pages that were all standardized so that all participants were presented with the same content. Next, participants were instructed on solving modular arithmetic problems, that each consisted of a term like the following:

$$ 29\, = \,11 \, ({\text{mod}}\,9) $$

The task is to subtract the number on the left side of the brackets (here: 11) from the number on the left side of the equals sign (here: 29) and indicate whether the result (here: 18) can be divided by the modus in brackets without fractions (here: yes). Participants were instructed to press a button labeled “richtig” (english: correct) for integral results and a button labeled “falsch” (english: false) for non-integral results. Each problem was presented for a maximum of 7 s, with a 500 ms blank in between. Most importantly, participants were informed that they would be interrupted randomly at several time points during the inquiry, having to switch to the context of modular arithmetic problem solving. They were also informed that this meant that they would either lose their work progress in the sidebar or that this progress would be reliably saved for later usage during the filling in of the fact sheet.

Each participant was randomly assigned to one of four versions of the experiment: in two versions, the first trial was a saved trial, in the other two versions the first trial was a deleted trial. Both fact sheets were equally often used in the first and second trial as well as in either saved trial or deleted trial.

**Figure 2:**
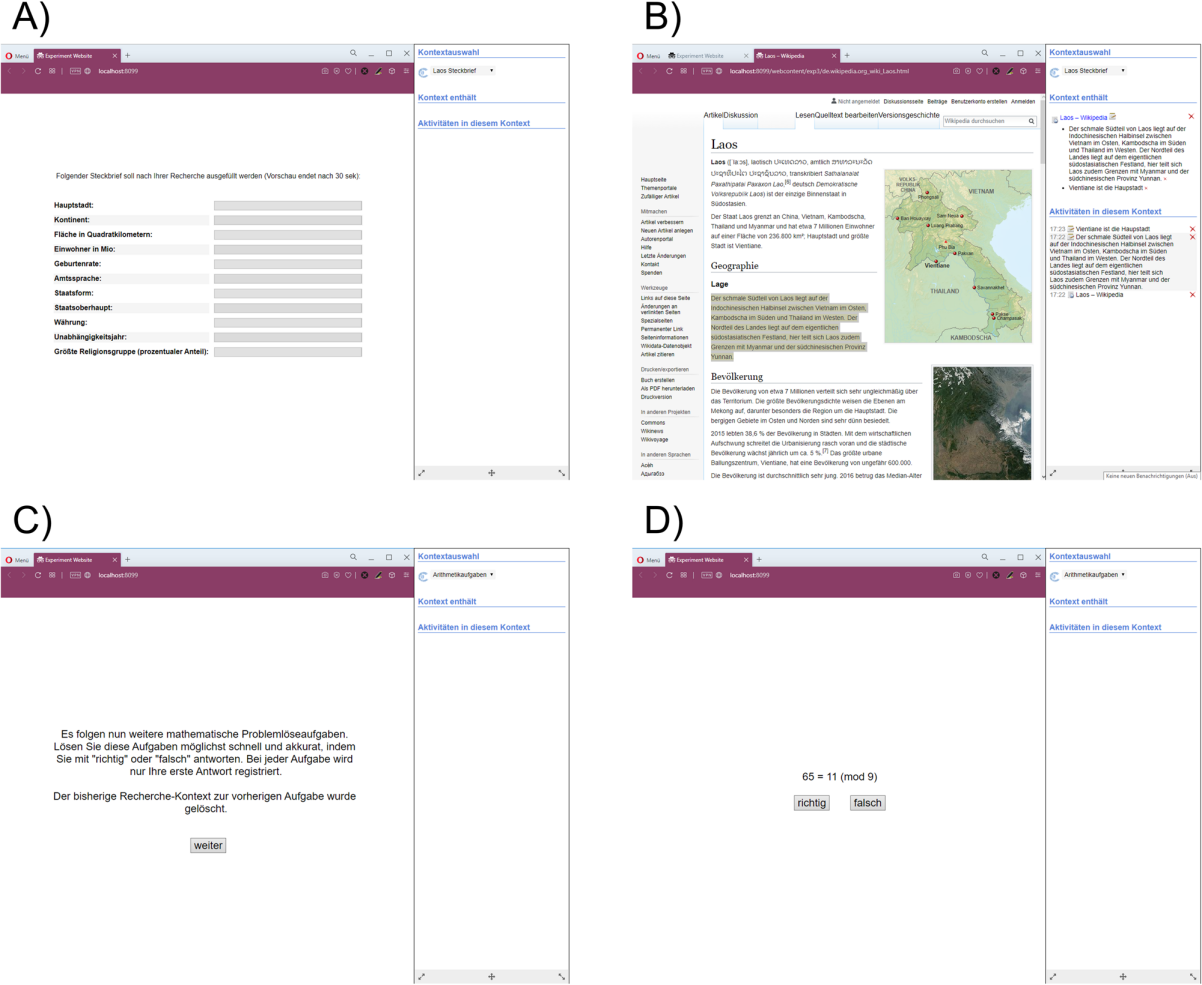
Setup of different experimental phases. (**A**) Fact sheet. (**B**) Inquiry. (**C**) Saving Cue. (**D**) Modular arithmetic problems.

The first trial started with a 30 s presentation of the first fact sheet (see Fig. 2A), whereupon participants engaged in their first inquiry (see Fig. 2B). Even though participants were made to believe that they would be interrupted at random time points, the first interruption always occurred after four minutes. A note appeared that instructed them to switch to the context of modular arithmetic problem solving by clicking on the “switch context”-button. After switching, all gathered content from the inquiry context disappeared from the sidebar. An additional note appeared, indicating that participants now had to solve modular arithmetic problems and informing them whether sidebar content had been saved for later use or deleted (see Fig. 2C). Next, participants solved 15 modular arithmetic problems without sidebar content visible (see Fig. 2D). No feedback was given on their performance. After this, they were asked to switch back to the inquiry context where they were given two minutes to start filling in the fact sheet for the first time—either with the help of the sidebar (saved trials) or by purely relying on their memory (deleted trials). Next, they continued their inquiry in a second round. In saved trials, previously saved content remained displayed in the sidebar. In deleted trials, the sidebar was empty, but could be used just like before. During the second inquiry, the interruption always occurred after three minutes, displaying the same note as before, meaning that in saved trials, content was saved after both inquiries (vice versa for deleted trials). Participants, however, were not aware of this fact. Again, this was followed by 15 modular arithmetic problems and a final phase of two minutes of continuing filling in the fact sheet. This was followed by one more trial with a new fact sheet. If the first trial had been a saved trial, the second trial was a deleted trial and vice versa.

### fNIRS measurement

An eight source, eight detector, portable, time-multiplexed, two wavelengths NIRSportTM (NIRx Medical Technologies LLC, USA) fNIRS device was used to record hemodynamic changes. Optodes were placed in a standardized, 10–10 NIRScaps™ (NIRx Medical Technologies LLC, USA). Optode placement was determined with the fNIRS Optodes' Location Decider (fOLDv2.2). fOLD is a Matlab (MathsWorks, USA) based toolbox which computes the optimal optode placement in the 10–10 system for covering specific brain areas. For optimal coverage of the MFG AF3, AF4, AF7, AF8, F3, F4, FC1 and FC2 were computed as source positions and FP1, FP2, F1, F2, F5, F6, FC3 and FC4 were computed as detector positions. This resulted in eighteen different channels each of which recorded the MFG with a specificity of 22.44% or greater. Signals were recorded with a frequency of 7.81 Hz. The NIRStar™ recording software (NIRx Medical Technologies LLC, USA) was used to digitalize signals.

### fNIRS data preprocessing and analysis

NIRS Brain AnalyzIR Toolbox^28^ was used to preprocess and subsequently analyze data. In a first step, raw voltage data was transformed into light-intensity data. This data was then used to calculate the relative concentration of oxygenated and deoxygenated hemoglobin via Beer-Lambert-Law^29^. The preprocessed data was subsequently entered into a two-level GLM. The analysis included four predictors, coding (1) neural activity during the first arithmetic block in the sidebar saved condition, (2) neural activity during the second arithmetic block in the sidebar saved condition, coding (3) neural activity during the first arithmetic block in the sidebar deleted condition, and (4) neural activity during the second arithmetic block in the sidebar deleted condition. Predictors for the GLM were generated by convolving each event with the canonical hemodynamic response function (HRF). First level analysis was conducted for each subject separately. To adapt modeling for individual differences in onset and dispersion of HRF, we included the first and second temporal derivative of each prediction term. To account for serially auto-correlated errors as well as artifacts induced by systemic physiology and motion, we applied an algorithm (AR-IRLS^29^) encompassing both pre-whitening and robust regression. For the second level analysis, the regression beta values obtained for each experimental condition for each subject were entered into a weighted mixed effects model. A fixed intercept for each experimental condition and a random intercept for each subject to best fit the overall data was estimated. For further analysis, two regions of interest (ROIs), left DLPFC (laDLPFC: AF7–FP1, AF7–F5, AF3–FP1, AF3–F5, F3–F5, F3–F1, F3–FC3, FC1–F1, FC1–FC3) and right DLPFC (AF8–FP2, AF8–F6, AF4–FP2, AF4–F6, F4–F6, F4–F2, F4–FC4, FC2–F2, FC2–FC4) where built as described in Santosa et al.^27^ (see Figure XY). Subsequently, differences in local blood flow between the sidebar saved and sidebar deleted conditions were compared via t-contrasts. We accounted for alpha inflation due to multiple comparisons by correcting all *p* values applying positive false discovery rate (FDR^30^). Only contrasts and correlations that yielded corrected *p* < 0.05 were regarded as statistically significant.

## Data Availability

Our data is publicly available via PsychArchives a service by the Leibniz Institute for Psychology Information (https://www.psycharchives.org/jspui/handle/20.500.12034/8065).

## Code availability

Our data is publicly available via PsychArchives a service by the Leibniz Institute for Psychology Information (https://www.psycharchives.org/jspui/handle/20.500.12034/8066).
